# Case-control study of hypertension and Parkinson’s disease

**DOI:** 10.1038/s41531-021-00202-w

**Published:** 2021-07-21

**Authors:** Yuen-Fann Ng, Ebonne Ng, Ee-Wei Lim, Kumar M. Prakash, Louis C. S. Tan, Eng-King Tan

**Affiliations:** 1 Duke-NUS Medical School, 8 College Road, Singapore, 169857 Singapore; 2 Department of Neurology, National Neuroscience Institute, 11 Jalan Tan Tock Seng, Singapore, 308433 Singapore

**Keywords:** Parkinson's disease, Parkinson's disease

## Abstract

We evaluate the association of hypertension with PD in an Asian population and performed a meta-analysis on similar studies to address the effect of hypertension on PD risk. A matched case-control study involving 1342 Chinese subjects (671 PD and 671 age and gender-matched controls (with a mean age of 63.9 ± 9.7 and 63.5 ± 9.8 years, and identical proportion of gender distribution) was conducted. Hypertension increases PD risk by 1.9 times [OR 1.86 (1.46–2.38)]. The literature search identified 618 studies initially; however, only three matched case-control studies (all in Caucasians) met the inclusion criteria for meta-analysis. Overall analysis showed that hypertension decreases PD risk by 0.2 times [OR 0.80 (0.66–0.96)]. Hypertension increases PD risk by 1.9 times in our Asian population. However, a meta-analysis comprising of Caucasian populations showed a protective effect of hypertension suggesting that ethnic differences or other genetic or environmental factors may contribute to the divergent observation. Early diagnosis and treatment of hypertension may potentially reduce the risk of PD, at least in our population.

## Introduction

Parkinson’s disease (PD) is a debilitating neurodegenerative disorder clinically characterized by resting tremor, bradykinesia, rigidity, gait disturbances, and postural instability. In addition to motor impairments, PD is associated with many non-motor features, such as constipation, lower urinary tract symptoms, sleep disturbances, and neuropsychiatric symptoms^1^. PD incidence increases with age, with a rate of 32 per 100 000 among individuals 50 years or above in the Singapore population comprising predominantly of ethnic Han Chinese^2^. The disease burden is expected to increase in rapidly ageing populations.

The pathophysiology behind PD is complex and likely to be influenced by the interplay of genetic and environmental factors^3^. Environmental risk factors that might predispose individuals to PD remain to be fully elucidated. Hypertension is one of the most common chronic diseases globally. In our population, about 20% aged 18−69 years had hypertension^4^. Hypertension is known to increase the risk of cardiovascular diseases^5^ and is one of the leading risk factors for cerebral small vessel disease, as demonstrated in human and animal studies^6,7^. Hypertension accounts for high risk of mortality and substantial disability^8^. Dietary and lifestyle changes can potentially improve blood pressure control, preventing hypertension, and decrease the risk of associated medical complications^5^.

The etiologic link between hypertension and PD has been debated^9^, with studies showing divergent results^10,11^. The neurodegenerative effect is postulated to be caused by vasculopathy leading to strategic infarcts that could affect dopaminergic cells and disrupt neuronal connections^12^. The vasculopathy pattern differs between Asians and Caucasians with Asians being more likely to suffer from small vessel strokes^13,14^. To date, there is a paucity of data on the association between hypertension and PD.

To address the gaps in current knowledge, we investigate the association between hypertension and PD risk utilizing a matched case-control study. In addition, we performed a pooled analysis of similar matched case-control studies to further explore the association of hypertension on PD risk.

## Results

### Hypertension and risk of PD

A total of 1342 subjects comprising of 671 PD and 671 age and gender-matched controls were included.

The mean age of cases and controls were 63.9 ± 9.7 and 63.5 ± 9.8 years respectively. Gender distribution was equal for both cases and controls, with 59.5% males and 40.5% females in each group. PD cases were more likely to have hypertension (46.1%) (Table 1). The proportion of smokers and non-smokers was equal in cases and controls. The presence of hypertension was significantly associated with a higher odds of PD risk compared to the absence of hypertension [OR 1.86 (1.46–2.38)] after adjusting for age and gender (Table 2). When further adjusted for potential vascular confounders including diabetes, hyperlipidemia, and smoking status, our results remained significant, [OR 1.52 (1.17–1.98)].

**Table 1:** Baseline characteristics of PD cases and controls.

Variable	PD (*n* = 671)	Controls (*n* = 671)	*P* value
*Demographics*			
Age (Years)	63.9 ± 9.72	63.5 ± 9.84	Matching factor
Male gender (%)	399 (59.5)	399 (59.5)	Matching factor
Hypertension (%)	309 (46.1)	214 (31.9)	<0.0001

**Table 2:** Summary of matched case-control studies on the association of hypertension with risk of Parkinson’s disease.

Study	Country	Sample size	Assessment of hypertension	OR (95% CI)	*P* value	Matching criteria
Current study	Singapore	671 PD, 671 controls	Questionnaire and medical record	1.86 (1.46–2.38)	<0.0001	Gender, age
Paganini-Hill^17^	USA	395 PD, 2320 controls	Questionnaire	0.71 (0.56–0.89)	None	Gender, age, vital status
Powers et al.^16^	USA	Males: 217 PD, 298 controls	Questionnaire	0.80 (0.55–1.17)	None	Gender, age, area
Becker et al.^15^	UK	3637 PD, 3637 controls	Medical record	0.83 (0.74–0.92)	0.001	Gender, age, general practice, index date, and duration of previous history

### Meta-analysis of matched case-control studies

**Fig. 1:**
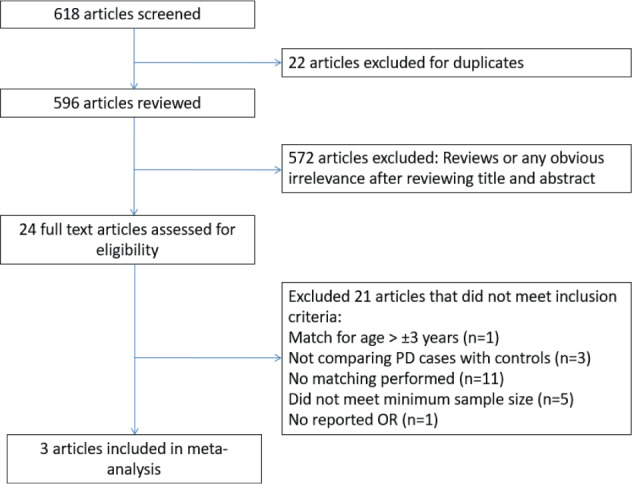
Selection for included studies in meta-analysis.

Our literature search identified an initial 618 studies in which 22 articles were duplicates and excluded. After the screening of the titles and abstracts, as well as assessment of eligibility criteria, only three studies with matched case-control samples, were included in the meta-analysis. The detailed selection process is shown in Fig. 1. The characteristics of included studies are shown in Table 2.

**Fig. 2:**
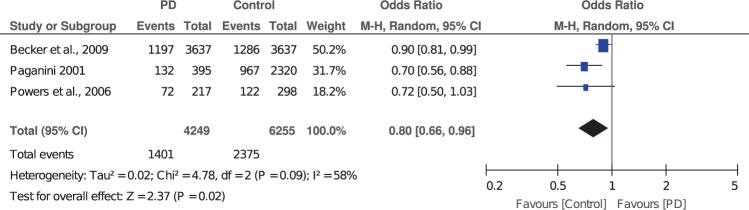
Forest plot of pooled analysis for matched case-control studies on the association of hypertension with risk of Parkinson’s disease. Individual and overall ORs and the corresponding 95% CIs are reported. The size of the box is proportional to the weight assigned to each study, and the horizontal lines represent the 95% CIs.

A meta-analysis of matched case-control studies showed a protective effect of hypertension on the risk of PD. The association was significant after adjustment for potential confounders [OR 0.80 (0.66–0.96)] (Fig. 2). There was considerable heterogeneity among studies in the pooled analysis (*I*^*2*^ = 58%).

## Discussion

Utilizing a closely matched case-control methodology, our study suggested that hypertension is associated with an increased PD risk in our Asian population. In comparison, pooled analysis of matched case-control studies, which comprised of Caucasian populations showed that hypertension is associated with reduced risk of PD.

Epidemiologic studies on hypertension generally had inconsistent associations with PD^9^. These discrepancies could be explained by heterogeneity in the study population and design. Although subgroup analysis or meta-regression was not performed, we observed that all three included studies had a similar effect, indicating an inverse relationship of hypertension with PD^15–17^. This could suggest that the source of heterogeneity might be due to variations in magnitude instead of the direction of effects. The heterogeneity could also arise from different study designs. The studies included had mostly different numbers of PD cases and controls. One study was a nested case-control study, which explained the relatively high sample size of 3637 PD and 3637 controls, compared to the other two case-control studies with varying sample sizes as well. Moreover, the studies have adjusted for different confounders, which further substantiate that the heterogeneity could come from the adjustment for different covariates. There were no Asian studies included in this meta-analysis as these studies did not meet our inclusion criteria. Either no matching was performed, or the focus of analysis was not between PD cases and controls.

In our analysis, all three published studies had a higher proportion of hypertension in the controls. Whereas in our study, a higher proportion was observed in PD cases. This divergent observation could be explained by a variance in prevalence of PD and hypertension in different populations, ethnic-specific genetic factors, or exposure to environmental agents or lifestyle habits, or a combination of these factors. Both PD and hypertension are chronic diseases with rising prevalence globally. It has been shown that the prevalence of PD is lower in Asian countries such as China and Japan, compared to a higher prevalence in Western populations^18^. A low prevalence of hypertension was seen in Koreans and Chinese compared with Caucasians^19,20^. However, certain Asian races such as Filipinos had a higher prevalence of hypertension^20^.

Interestingly, some studies, which did not satisfy our meta-analysis inclusion criteria showed that hypertension was significantly associated with increased risk of PD among Asians^11^, corroborating our observation. If we have included studies with non-matched samples in our meta-analysis, there is a shift in trend towards higher odds of PD risk in the presence of hypertension (data not shown). Multiple non-matched Western studies have also established that hypertension increases PD risk^12,21,22^. In the Asian population, cohort studies in Taiwan showed an increased risk of PD in the presence of hypertension^23,24^, this is also seen in the Korean population^25^. However, a study in Japan showed that hypertension is associated with a decreased risk of PD^26^. These differences suggest potential underlying heterogeneity and subgroup variation, which could be attributed to many factors such as ethnic-specific genes or differences in lifestyle habits.

Emerging studies suggest that hypertension might share similar pathogenic pathways with PD, although exact mechanisms remain to be elucidated and require further investigations from in-vitro and animal models. The advantageous effect of hypertension on PD was postulated to be due to persistent elevation of cerebral blood flow^16^. Given that hypertension is one of the major risk factors of cerebral small vessel disease and has shown to contribute to cognitive decline and Alzheimer’s disease^6^, it is highly plausible that hypertension could result in greater PD risk. Chronic hypertension leading to infarcts at strategic locations such as the basal ganglia could disrupt dopaminergic cells and neuronal connections, subsequently leading to cerebrovascular dysfunction as suggested by pathological and imaging studies^27,28^. Furthermore, long-standing hypertension increases the risk of oxidative stress, resulting in reduced cerebral perfusion, which could decrease oxygen delivery to brain regions implicated in PD that are highly susceptible to ischemia^29,30^.

It has been suggested that reduced sympathetic activity in PD, due to decreased in levels or blunted activity of hormones involved in the sympathetic nervous system, could possibly explain the inverse relationship between hypertension and PD^31^. PD-induced harmful alterations to the autonomic nervous systems were shown. However, in addition to the sympathetic nervous system, the parasympathetic function is impaired as well. This results in the decreased ability to regulate blood pressure changes leading to a higher ambulatory blood pressure variability in PD^32^. A greater understanding of the pathogenesis pathways would be necessary to form the basis for the future development of pharmacological interventions in PD.

Our study involved a closely matched case-control data set. In addition, as Asian ethnicity may be varied (just like Caucasian race), we have only included ethnic Han Chinese, thus reducing racial difference as a confounding factor. It is unclear if our findings could be extrapolated to other populations since the underlying gene and environmental exposures may be ethnic-specific. Further studies in well-designed large prospective studies to better understand the interplay of environmental and gene factors, and the pathogenic mechanisms of PD are warranted. We have included matched studies with at least 200 cases and 200 controls in our meta-analysis because small unmatched studies are at a higher risk of both type 1 and type 2 errors. Future matched case-control studies in the Asian populations should be performed to corroborate our findings.

There is potential for our findings to contribute to the institution of behavioral modification interventions including lifestyle modifications or dietary recommendations for PD patients and asymptomatic high-risk populations to mitigate disease risk. Studies at the molecular level can be used to investigate the effects of hypertension on PD. These will be essential in elucidating the shared biomolecular mechanisms underlying both chronic diseases. A better understanding of the pathophysiology could form the basis for the future development of individualized disease-modifying therapies in PD^33^.

We demonstrated that hypertension increases PD risk by 1.9 times in our Asian population. However, our meta-analysis comprising studies in Caucasian populations was unable to replicate the finding suggesting that ethnic differences may contribute to divergent observation. Future longitudinal studies to evaluate the role of ethnic-specific gene variants in mediating the interaction between hypertension and PD will be of interest. Our study also suggests that early diagnosis and treatment of hypertension may potentially reduce the risk of PD, at least in our population.

## Methods

### Study subjects

Patients diagnosed with PD by movement disorder neurologists according to the UK PD Society Brain Bank clinical diagnostic criteria^34^ were recruited as cases at two major movement disorders centers. Healthy controls were volunteers without neurodegenerative diseases and examined by the investigators. We have only included ethnic Han Chinese in our study. For every PD patient, we looked for a similar age (± 2 years) and gender-matched control subjects.

The study received approval from the Singapore General Hospital/Singhealth institutional ethics committee (2002/08/A). Subjects have given written informed consent. The methods were carried out in accordance with the approved guidelines.

### Assessment of hypertension

We used a previously validated questionnaire to obtain clinical information on hypertension, demographics, and family history of movement disorders from participants^35^. A response was coded as “No” if the subject never had hypertension and “Yes” if the subject was diagnosed with the condition by their physicians, supported by the use of prescribed medications. In addition to self-reported blood pressures, objective measurement was performed in clinics during recruitment. Subjects with high blood pressures during the clinical examination were subsequently diagnosed with hypertension and classified as hypertension for the purpose of this study.

### Literature search and eligibility criteria

A database search using PubMed from the last 20 years (2000–2020) was performed. The following search terms were used: (“Parkinson’s disease” or “Parkinson disease”) and (“hypertension” or “high blood pressure”). Studies that met the following eligibility criteria were included for meta-analysis. Inclusion criteria included: (1) publications limited to English and human subjects only; (2) at least 200 cases and 200 controls comparing hypertension between PD cases and controls; (3) age and gender must be closely matched in cases and controls (age difference of at most ±3 years); (4) available data on the frequency of hypertension, age, and gender in cases and controls; (5) assessment of hypertension was provided; (6) PD diagnosis criteria were provided; (7) available data on odds ratio (OR), relative risk (RR) or hazard ratio (HR) and 95% confidence interval (CI).

Studies were excluded based on the following criteria: (1) studies without original data such as reviews or letters; (2) not matched for both age and gender; (3) wide age difference in matching (> ±3 years); (4) OR, RR or HR, and 95% CI were not provided or could not be derived from calculations based on available data.

### Statistical methods

Subjects with demographic data for age, gender, and hypertension were included in the analysis. Baseline characteristics were reported using descriptive statistics and were compared between PD and controls using McNemar test. OR with 95% CI were derived from conditional logistic regression models adjusted for age and gender to study the independent effect of hypertension with PD risk. In the meta-analysis, a random-effects model was used to derive pooled OR with 95% CI given *I*_2_ > 50%. Subgroup analysis and meta-regression were not performed given the small number of available studies^36^. The significance level was set at *p* < 0.05. An *I*_2_ value that is more than 50% would indicate the presence of significant heterogeneity^37,38^.

Data analysis was performed using SPSS (IBM Corp. Released 2010. IBM SPSS Statistics for Windows, Version 19.0. Armonk, NY: IBM Corp.). Meta-analysis was performed usingReview Manager (RevMan) [Computer program]. Version 5.4.1 The Cochrane Collaboration, 2020.

### Reporting summary

Further information on research design is available in the Nature Research Reporting Summary linked to this article.

## Supplementary information

Reporting Summary

## Data Availability

The datasets analyzed during the current study are available from the corresponding author on reasonable request.
