# Supplementary material for: Case-control study of hypertension and Parkinson’s disease
Source: NPJ Parkinsons Dis. 2021 Jul 21;7:63. doi: 10.1038/s41531-021-00202-w (PMC8295270; doi:10.1038/s41531-021-00202-w)
Supplement: Supplementary file 1 — Reporting Summary [file 41531_2021_202_MOESM1_ESM.pdf]

nature research

Corresponding author(s): Eng-King Tan

Last updated by author(s): Feb 21, 2021

Reporting Summary

Nature Research wishes to improve the reproducibility of the work that we publish. This form provides structure for consistency and transparency in reporting. For further information on Nature Research policies, see our Editorial Policies and the Editorial Policy Checklist.

Statistics

For all statistical analyses, confirm that the following items are present in the figure legend, table legend, main text, or Methods section.

n/a	Confirmed	
☐	☒	The exact sample size (*n*) for each experimental group/condition, given as a discrete number and unit of measurement
☐	☒	A statement on whether measurements were taken from distinct samples or whether the same sample was measured repeatedly
☐	☒	The statistical test(s) used AND whether they are one- or two-sided<br>*Only common tests should be described solely by name; describe more complex techniques in the Methods section.*
☐	☒	A description of all covariates tested
☒	☐	A description of any assumptions or corrections, such as tests of normality and adjustment for multiple comparisons
☐	☒	A full description of the statistical parameters including central tendency (e.g. means) or other basic estimates (e.g. regression coefficient) AND variation (e.g. standard deviation) or associated estimates of uncertainty (e.g. confidence intervals)
☐	☒	For null hypothesis testing, the test statistic (e.g. *F*, *t*, *r*) with confidence intervals, effect sizes, degrees of freedom and *P* value noted<br>*Give P values as exact values whenever suitable.*
☒	☐	For Bayesian analysis, information on the choice of priors and Markov chain Monte Carlo settings
☒	☐	For hierarchical and complex designs, identification of the appropriate level for tests and full reporting of outcomes
☒	☐	Estimates of effect sizes (e.g. Cohen's *d*, Pearson's *r*), indicating how they were calculated

Our web collection on statistics for biologists contains articles on many of the points above.

Software and code

Policy information about availability of computer code

Data collection	No software was used
Data analysis	Data analysis was performed using SPSS (IBM Corp. Released 2010. IBM SPSS Statistics for Windows, Version 19.0. Armonk, NY: IBM Corp.). Meta-analysis was performed usingReview Manager (RevMan) [Computer program]. Version 5.4.1 The Cochrane Collaboration, 2020.

For manuscripts utilizing custom algorithms or software that are central to the research but not yet described in published literature, software must be made available to editors and reviewers. We strongly encourage code deposition in a community repository (e.g. GitHub). See the Nature Research guidelines for submitting code & software for further information.

Data

Policy information about availability of data

All manuscripts must include a data availability statement. This statement should provide the following information, where applicable:

- Accession codes, unique identifiers, or web links for publicly available datasets
- A list of figures that have associated raw data
- A description of any restrictions on data availability

The datasets analysed during the current study are available from the corresponding author on reasonable request

Field-specific reporting

Please select the one below that is the best fit for your research. If you are not sure, read the appropriate sections before making your selection.

☐ Life sciences ☒ Behavioural & social sciences ☐ Ecological, evolutionary & environmental sciences

For a reference copy of the document with all sections, see nature.com/documents/nr-reporting-summary-flat.pdf

Behavioural & social sciences study design

All studies must disclose on these points even when the disclosure is negative.

Study description	A matched case-control study to investigate the association between hypertension and PD risk. In addition, a pooled analysis of similar matched cases-control studies to further explore the association of hypertension on PD risk.
Research sample	For every PD patient, we looked for a similar age (±2 years) and gender matched control subject. A total of 1342 subjects comprising of 671 PD and 671 age and gender matched controls were included.
Sampling strategy	For every PD patient, we looked for a similar age (±2 years) and gender matched control subject. A total of 1342 subjects comprising of 671 PD and 671 age and gender matched controls were included.
Data collection	We used a previously validated questionnaire to obtain clinical information on hypertension, demographics and family history of movement disorders from participants. A response was coded as ''No'' if the subject never had hypertension and ''Yes'' if the subject was diagnosed with the condition by their physicians, supported by the use of prescribed medications. In addition to self-reported blood pressures, objective measurement was performed in clinics during recruitment. Subjects with high blood pressures during clinical examination were subsequently diagnosed with hypertension and classified as hypertension for the purpose of this study.
Timing	Our subjects were recruited from 2000 to April 2020. A database search using PubMed from the last 20 years (2000-2020) was performed.
Data exclusions	Only Han Chinese was included in our study to reduce ethnic difference as a confounding factor.<br><br>In our meta-analysis, Studies that met the following eligibility criteria were included for meta-analysis. Inclusion criteria included: 1) Publications limited to English and human subjects only; 2) At least 200 cases and 200 controls comparing hypertension between PD cases and controls; 3) Age and gender must be closely matched in cases and controls (age difference of at most ±3 years); 4) Available data on frequency of hypertension, age and gender in cases and controls; 5) Assessment of hypertension was provided; 6) PD diagnosis criteria was provided; 7) Available data on OR, RR or HR and 95% CI.<br><br>Studies were excluded based on the following criteria: 1) Studies without original data such as reviews or letters. 2) Not matched for both age and gender; 3) Wide age difference in matching (>±3 years); 4) OR, RR or HR and 95% CI were not provided or could not be derived from calculations based on available data; 4) Studies without original data such as reviews or letters.
Non-participation	No participants dropped out
Randomization	No randomization was performed

Reporting for specific materials, systems and methods

We require information from authors about some types of materials, experimental systems and methods used in many studies. Here, indicate whether each material, system or method listed is relevant to your study. If you are not sure if a list item applies to your research, read the appropriate section before selecting a response.

Materials & experimental systems

n/a	Involved in the study	
☒	☐	Antibodies
☒	☐	Eukaryotic cell lines
☒	☐	Palaeontology and archaeology
☒	☐	Animals and other organisms
☐	☒	Human research participants
☐	☒	Clinical data
☒	☐	Dual use research of concern

Methods

n/a	Involved in the study	
☒	☐	ChIP-seq
☒	☐	Flow cytometry
☒	☐	MRI-based neuroimaging

Human research participants

Policy information about studies involving human research participants

Population characteristics	A total of 1342 subjects comprising of 671 PD and 671 age and gender matched controls were included. The mean age of cases and controls were 63.9±9.7 and 63.5±9.8 years respectively. Gender distribution was equal for both cases and controls, with 59.5% males and 40.5% females in each group. PD cases were more likely to have hypertension (46.1%).
Recruitment	In a case-control study, our subjects were recruited from 2000 to April 2020. Patients diagnosed with PD by movement disorder neurologists according to the UK PD society Brain Bank clinical diagnostic criteria were recruited as cases at two major movement disorders centers (Singapore General hospital and Tan Tock Seng Hospital, National Neuroscience institute). Healthy controls without neurodegenerative diseases were recruited examined by investigators and recruited from a Community Healthy Screening Programme. We have only included ethnic Han Chinese in our study.
Ethics oversight	The study received approval from the Singapore General Hospital/Singhealth institutional ethics committee. Subjects have given written informed consent. The methods were carried out in accordance with the approved guidelines.

Note that full information on the approval of the study protocol must also be provided in the manuscript.

Clinical data

Policy information about clinical studies

All manuscripts should comply with the ICMJE guidelines for publication of clinical research and a completed CONSORT checklist must be included with all submissions.

Clinical trial registration	Not a clinical trial
Study protocol	The study protocol is available from the corresponding author on reasonable request
Data collection	We used a previously validated questionnaire to obtain clinical information on hypertension, demographics and family history of movement disorders from participants. A response was coded as ‘’No’’ if the subject never had hypertension and ‘’Yes’’ if the subject was diagnosed with the condition by their physicians, supported by the use of prescribed medications. In addition to self-reported blood pressures, objective measurement was performed in clinics during recruitment. Subjects with high blood pressures during clinical examination were subsequently diagnosed with hypertension and classified as hypertension for the purpose of this study.
Outcomes	we investigate the association between hypertension and PD risk utilizing a matched case-control study. In addition, we performed a pooled analysis of similar matched cases-control studies to further explore the association of hypertension on PD risk.
